# ASO Author Reflections: Biopsy Guided Pathological Response Assessment in Breast Cancer is Insufficient: Additional Pathology Findings of the MICRA Trial

**DOI:** 10.1245/s10434-023-13546-9

**Published:** 2023-05-18

**Authors:** Annemiek K. E. van Hemert, Frederieke H. van Duijnhoven, Marie-Jeanne T. F. D. Vrancken-Peeters

**Affiliations:** 1 Departments of Surgical Oncology, Netherlands Cancer Institute - Antoni van Leeuwenhoek, Amsterdam, The Netherlands; 2 Department of Surgery, Amsterdam University Medical Center, Amsterdam, The Netherlands

## Past

Since the introduction of neoadjuvant systemic therapy (NST), there are increasing possibilities to de-escalate locoregional treatment. The usage and effectiveness of NST are on the rise, with high pathological complete response (pCR) rates observed, particularly in patients with triple-negative (TN) and HER2-positive breast cancer.^1–3^ It remains unclear whether breast surgery contributes to local control and survival in patients who achieve pCR. However, although various studies have investigated minimally invasive techniques for predicting response to NST, no technique has yet showed an acceptable false negative rate (FNR) for predicting pCR for the entire study cohort.^4–8^ The studies differed in radiologic response assessment and biopsy technique. Optimal radiologic assessment with magnetic resonance imaging (MRI), used in the MICRA trial, was associated with a higher FNR.^9^ Studies where more tissue was obtained with larger or > 12 biopsies showed lower FNRs.^6,8^

## Present

Although the exact acceptable FNR for detection of pCR after NST is still under debate, as adjuvant systemic treatment improved survival in patients with (any) residual invasive disease,^10,11^ several attempts are made to define a subgroup of excellent responders in which an acceptable FNR can be achieved.^4,12–14^ These results should be interpreted with caution, as the studies were not powered for post hoc subgroup analyses. Another approach in the attempt of lowering the FNR is the introduction of machine-learning techniques incorporating patient, imaging, tumor, and biopsy characteristics of previously conducted studies.^12,14–17^ While with an obtained FNR of only 0% this is a promising approach, further investigation is needed to determine its applicability in the clinical setting. Meanwhile, one trial is currently investigating the oncologic outcome of omission of surgery in patients with a pCR post-NST on the basis of vacuum assisted biopsies (VAB).^18^ Two-year follow-up of 31 participating patients showed no local recurrences.^18^ It should be noted that a mean of 15 VAB (9G) are obtained, and one might question whether this can be considered as a minimally invasive technique.

## Future

Accurate assessment of the response to NST is of importance, especially in patients with TN and HER2-positive breast cancer, as they have a survival benefit from adjuvant systemic treatment in case of residual invasive disease.^10,11^ Although surgery may not attribute to local control or survival, it is still essential as diagnostic tool in these patients. In our opinion, the low risk of complications and morbidity associated with a diagnostic lumpectomy, where < 10 grams of tissue is removed, outweighs the accuracy of residual disease assessment in order not to withhold patients from adjuvant systemic treatment. Our pathology analysis of the MICRA study shows that limited but relevant residual invasive disease was missed in patients with TN or HER2-postive breast cancer when surgery was omitted.^19^ Therefore, it is time to consider alternative de-escalation strategies, such as omission of radiotherapy instead of surgery. The DESCARTES trial (De-ESCAlating RadioTherapy in breast cancer patients with pathologic complete response to neoadjuvant systemic therapy, NCT05416164) aims to show the safety of omission of radiation treatment in cT1–2N0 patients achieving pCR after NST, detected with surgery.^20^
